# Endovascular management of primary long-segment inferior vena cava occlusion: treatment strategies and clinical outcomes

**DOI:** 10.3389/fphar.2025.1512157

**Published:** 2025-06-06

**Authors:** Jianjun Jiang, Mingli Li, Guangzhen Li, Yang Liu, Qingbo Su

**Affiliations:** ^1^ Department of Vascular Surgery, Qilu Hospital of Shandong University, Jinan, China; ^2^ Department of Vascular Surgery, Qilu Hospital of Shandong University, Cheeloo College of Medicine, Shandong University, Qingdao, China

**Keywords:** inferior vena cava, long-segment occlusion, endovascular treatment, technical success, patency rate, local anesthesia, stent placement, color Doppler ultrasound

## Abstract

**Background:**

Primary long-segment occlusion of the inferior vena cava (IVC) is a rare condition with diverse clinical presentations. The optimal management approach for this condition remains uncertain, warranting further investigation into endovascular treatment methods.

**Methods:**

A retrospective study conducted at Qilu Hospital of Shandong University from 2012 to 2018 assessed 16 patients with primary long-segment IVC occlusion. Patients underwent comprehensive imaging evaluations and received endovascular interventions such as angioplasty, stent placement, and online blood flow restoration. Additionally, a literature review was performed to analyze current practices in managing IVC occlusion.

**Results:**

Endovascular treatment was completed in all patients, with favorable primary and secondary patency rates during follow-up. Clinical symptoms significantly improved post-treatment, and the majority of patients achieved IVC patency without major complications. The study showcased the efficacy of angioplasty and stent placement in managing primary IVC occlusion.

**Conclusion:**

Endovascular therapy is a safe and effective approach for tackling long-segment IVC occlusion, leading to improved patient outcomes. Long-term anticoagulant prophylaxis is advised to mitigate the risk of venous thrombosis in these patients. This study contributes valuable insights for guiding clinical practice in treating primary IVC occlusion.

## Introduction

The inferior vena cava (IVC) is responsible for returning deoxygenated blood from the lower body to the heart, making it the most prominent vein in the human body. The healthy functioning of the IVC is crucial for maintaining the balance of the circulatory system (Alkhouli et al., 2016). However, occlusion of the IVC often occurs in long segments and can be caused by various factors such as tumor compression, venous thrombosis, congenital interruption of IVC, inflammation, trauma, and infection (Missal et al., 1965; Razavi et al., 2000; Raju et al., 2006; te Riele et al., 2006; Sitwala et al., 2014; Kuetting et al., 2017). Although rare, the etiology and impact of IVC occlusion still need to be fully understood. This occlusion can lead to blocked blood reflux, resulting in symptoms such as abdominal pain, lower limb edema, and varicose veins (Naqvi et al., 2016; Ai et al., 2022). As the disease progresses, patients may experience more severe complications, highlighting the need for prompt treatment (Rao et al., 2012).

Treatment options for long-segment occlusion of the IVC have been extensively studied. These options include drug therapy, surgery, and endovascular interventions (Wang et al., 2021; Bettiol et al., 2023). Conservative treatments such as compression therapy, leg elevation, and anticoagulation are commonly used but are often not sustainable long-term. Surgery is also not the preferred choice due to its associated early morbidity and long-term prognosis concerns (Gloviczki et al., 1992; Jost et al., 2001; Garg et al., 2011). As a result, selecting the most appropriate treatment strategy for IVC long-segment occlusion remains challenging. The rarity of this disease further complicates treatment decisions due to limited clinical experience. Moreover, potential complications and a high treatment failure rate concern physicians and patients (Ruiz et al., 2020). Therefore, there is an urgent need for more effective and safe treatment methods to improve patients’ quality of life and prognosis.

Endovascular therapy has emerged as a viable and effective treatment option for various venous diseases over the last decade (Hartung et al., 2005; DeRubertis et al., 2013; Erben et al., 2018). While previous studies have primarily focused on short-segment IVC occlusions caused by secondary factors like thrombosis or tumor compression (Raju et al., 2006; Kuetting et al., 2017), there remains limited research on the etiology of primary long-segment IVC occlusions (Rikitake et al., 2006; Lorenz et al., 2014; Zhang et al., 2015).

This study aims to comprehensively investigate the effectiveness of endovascular treatment methods for primary long-segment occlusion of the IVC. By retrospectively analyzing data from 16 patients treated at Qilu Hospital of Shandong University over 6 years, our objective is to provide more definitive and influential guidance for treating this rare disease. Furthermore, this study strives to advance research in this field and establish more dependable treatment options for clinical practitioners and patients.

## Materials and methods

### Study design and ethical approval

This retrospective study was conducted at Qilu Hospital of Shandong University to evaluate the effectiveness of endovascular treatment in patients with primary long-segment IVC occlusion. Ethical approval was obtained from the hospital’s Research Ethics Committee. All patients provided written informed consent after being fully informed of the study’s objectives and risks.

### Patient screening

Assessment of medical background and potential etiology included a review of relevant family history and factors such as pre-existing conditions, trauma, prior surgeries, oral contraceptive use, and venous thromboembolism. Patients treated between January 2012 and December 2018 were included if they met the following criteria: 1) diagnosed with primary long-segment IVC occlusion; 2) occlusion length ≥6 cm; and 3) presence of related clinical symptoms. Exclusion criteria included: 1) IVC compression due to tumor or thrombus; 2) age <18 years; and 3) occlusion caused by trauma, infection, or congenital abnormalities.

### Clinical assessment and diagnosis

All patients underwent preoperative computed tomography (CT) angiography to assess venous lesions, identify the location and extent of IVC occlusion, and exclude tumors or thrombosis. Color Doppler ultrasound was used to evaluate lower extremity deep venous thrombosis. Clinical status was documented using the CEAP classification and venous clinical severity score (VCSS).

### Risk assessment for blood clot formation

A comprehensive panel of blood tests was performed on all patients to assess their susceptibility to developing blood clot formation. The tests included evaluations for deficiencies in protein C, protein S, and antithrombin III and the presence of antinuclear antibodies, antiphospholipid antibodies, coagulation factors, homocysteine levels, and Factor V Leiden mutation.

### Patient preparation and drug pretreatment

Drug pretreatment: Upon admission to the hospital, all patients are prescribed low molecular weight heparin therapy at a dosage of 1 mg/kg, administered every 12 h. This treatment strategy aims to attain optimal anticoagulation effects in advance, ultimately mitigating the risk of thrombotic complications during the surgical procedure.

### Surgical procedure

**FIGURE 1:**
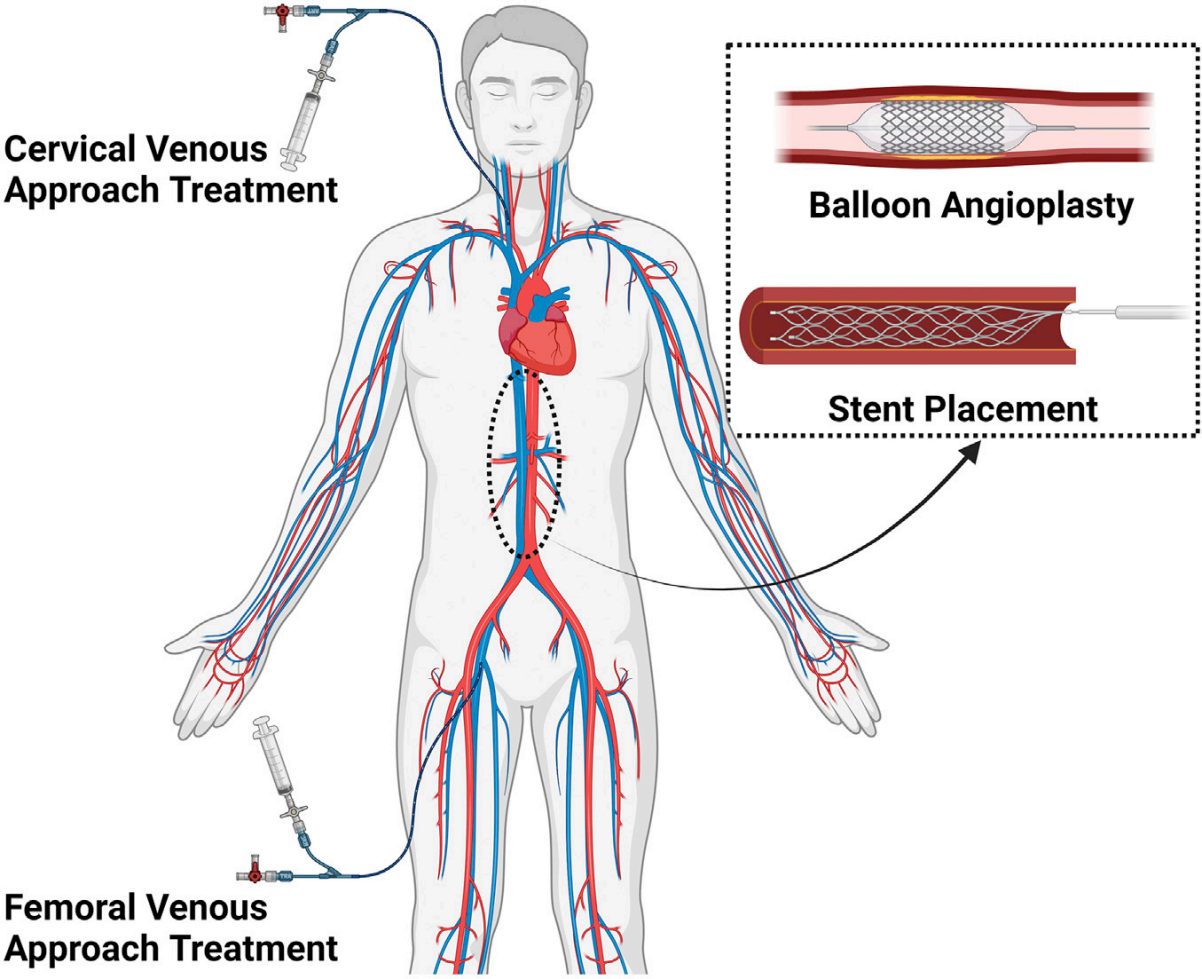
Illustration of endovascular treatment for primary long-segment occlusion of the IVC.

As illustrated in Figure 1, the endovascular procedure involves accessing the occluded inferior vena cava via the femoral or jugular vein, followed by balloon angioplasty and stent implantation to restore venous patency.

#### Positioning and anesthesia

Patients are placed in the supine position under local anesthesia without sedation.

#### Surgical approach

The choice of access is based on the occlusion site and patient condition. Bilateral femoral veins are preferred; the right jugular vein is used if femoral access is unavailable.

#### Anticoagulation management

Preoperative anticoagulation is maintained with unfractionated heparin to achieve an activated clotting time of 220–300 s, reducing intraoperative thrombosis risk.

#### Guidewire and catheter selection

When advancing the guide wire and catheter, priority should be given to hydrophilic guide wires, long introducer sheaths, and support catheters, especially for chronic occlusions. The support catheter from Cook (Bloomington, USA) is more accessible to advance compared to the sliding catheter from Terumo, Vietnam. A stiffer guide wire from Terumo, Vietnam, is used for continuous angioplasty and stent deployment.

#### Angioplasty and stent placement

Balloon dilation was performed using balloons with diameters ranging from 8 to 20 mm at inflation pressures of 8–12 atm to gradually expand the occluded segments. Stent implantation was indicated when residual stenosis after balloon dilation exceeded 30%. Self-expanding stents were used, with an overlap of more than 1 cm to ensure full coverage of the fibrotic segments. The inferior margin of the IVC stent was positioned at the iliac vein confluence. Stents were extended to cover the renal veins for supra-renal and infra-renal IVC occlusions. A 2 cm double-barrel “kissing stent” technique was commonly used to reconstruct the iliac bifurcation. Balloons used for stent deployment were 1–2 mm smaller in diameter than the corresponding stents.

Stent models and manufacturers included Wallstents (Boston Scientific, United States), Z-stents (Shenyang Yongtong Technology Co., Ltd., China), Zilver stents (Cook Medical, Ireland), and lumbar stents (Bard, Germany). Stent types and configurations were selected intraoperatively based on lesion extent and venous anatomy. In some cases, both iliac and IVC stents were combined for complete reconstruction.

### Postoperative management

Pharmacological treatment: Following surgery, it is recommended to administer low molecular weight heparin (1 mg/kg, every 12 h) as an anticoagulation therapy to maintain vascular patency. For long-term post-discharge management, patients should take oral warfarin or rivaroxaban. If using warfarin, the international normalized ratio should be maintained within the range of 2.0–3.0.

Compression therapy: To prevent venous complications after surgery, patients are advised to wear compression stockings above the knees with a pressure level of two–3. This should be implemented during surgery and continued after discharge.

Follow-up and re-intervention: Regular follow-up examinations, including CT angiography or color Doppler ultrasound, should be conducted 1, 3, 6, and 12 months post-operation to ensure venous access patency. In cases where new narrowings, occlusions, or thrombosis are detected during follow-up, timely re-intervention should be performed.

### Standards for technical success

This study defines technical success as the effective restoration of blood flow in patients following vascular angioplasty and stent placement while ensuring a residual stenosis rate of no more than 20%. This criterion is crucial for maintaining patients’ venous access and achieving the desired therapeutic outcome (Mahnken et al., 2014).

### Literature review data sources and retrieval strategies

To explore the advancements in endovascular treatment for patients with primary long-segment occlusion of the IVC, we conducted an extensive literature search on the topic of “IVC occlusion” using databases such as PubMed, MEDLINE, Embase, and Cochrane up until October 2023. These comprehensive literature sources serve as our primary data reference.

### Literature screening and quality assessment

Studies were included if they met the following criteria: 1) diagnosis of IVC occlusion; 2) occlusion caused by non-malignant tumors or thrombosis; 3) relevance to endovascular treatment. Exclusion criteria were letters, reviews, meta-analyses, non-human studies, and non-English publications.

The literature screening process used the reference management program EndNote X9 (Clarivate Analytics, United States). First, duplicate articles were automatically removed using the software; then, a manual check was performed. Two researchers independently screened the remaining articles based on titles, abstracts, and full-text content by pre-defined eligibility criteria (Castillo-Lopez et al., 2023). Any discrepancies were resolved through discussions with a third researcher (Liu et al., 2023). In cases of overlap between the study populations and the stents investigated, studies with a more significant number of participants were prioritized. Informed consent was obtained from all participants included in the studies. Furthermore, the methodological quality of the included studies was independently assessed by two researchers using Review Manager v5.4 software. Any discrepancies were resolved through discussions with a third researcher (Tang et al., 2022).

### Statistical analysis

The statistical analysis was conducted using IBM SPSS Statistics software (version 20.0; SPSS Inc., Chicago, IL, United States). The variables were expressed as the mean ± standard deviation (SD). We considered a p-value of less than 0.05 as statistically significant. We utilized Kaplan-Meier analysis to assess patency of the target vessels/stents during the follow-up period.

## Results

### Analysis of the efficacy and clinical features of endovascular treatment in patients with IVC occlusion

The clinical characteristics of the patients are summarized in Table 1. This study included various types of IVC occlusion: 2 patients had complete IVC occlusion, 6 had suprarenal segment occlusion, 4 had infrarenal occlusion, and 4 had both suprarenal and infrarenal involvement. Additionally, 6 patients had concurrent iliac vein occlusion.

**TABLE 1:** Clinical data of patients with long-segment of IVC occlusion.

No.	Age/Sex	Weight (kg)/height (cm) and BMI	Symptom	Comorbidity and risk factor	Occlusion location (occlusion length, cm)	CEAP/VCSS score		Treatment	Second intervention
						Pre-operation	Final follow-up		
1	50/M/	74/168/26.2	Abdominal varices and distention, bilateral lower extremity ulcerations, breast development	Smoke and alcohol	Total IVC and bilateral CIV obstruction (26)	C6/13	C5/7	IVC and right CIV stenting	-
2	49/F	73/165/26.8	Abdominal varices and distention, bilateral lower extremity ulcerations	-	Total IVC, right innominate vein and bilateral CIV (28)	C6/18	C5/8	Balloon angioplasty	-
3	28/M	83/181/25.3	Abdominal distention	-	Suprarenal IVC (6)	C1/1	C1/1	Balloon angioplasty	Second: IVC stenting Third: CDT and Balloon angioplasty
4	53/F	61/162/23.2	Abdominal and bilateral lower extremity varices, ulcerations	-	Infrarenal IVC and bilateral CIV (8)	C6/12	C5/7	IVC and bilateral CIV stenting	-
5	45/M	72/175/23.5	Abdominal and bilateral lower extremity varices	-	Suprarenal IVC (7)	C3/7	C1/3	IVC stenting	-
6	58/M	77/171/26.3	Abdominal and bilateral lower extremity varices	Smoke and alcohol	Suprarenal and infrarenal IVC(16)	C4/11	C2/4	IVC stenting	-
7	47/M	68/172/23.0	Bilateral lower extremity swelling and ulcerations	Smoke and alcohol	Infrarenal IVC and bilateral CIV (7)	C6/12	C5/7	IVC and bilateral CIV stenting	-
8	42/M	74/181/22.6	Abdominal and bilateral lower extremity varices	-	Suprarenal IVC (8)	C2/6	C2/2	IVC stenting	-
9	45/M	79/177/25.2	Abdominal and bilateral lower extremity varices	Smoke and alcohol	Suprarenal IVC (7)	C3/8	C2/3	IVC stenting	-
10	51/M	67/172/22.6	bilateral lower extremity varices	diabetes	Suprarenal and infrarenal IVC (15)	C2/6	C2/2	IVC stenting	-
11	37/F	60/158/24.0	Abdominal distention and pain	-	Suprarenal IVC (6)	C1/1	C1/1	IVC stenting	-
12	49/F	54/160/21.1	Bilateral lower extremity swelling ulcerations	hypertension	Infrarenal IVC and bilateral CIV (9)	C6/13	C5/7	IVC stenting and CIV balloon angioplasty	Balloon angioplasty and CDT
13	52/M	71/174/23.5	Abdominal and bilateral lower extremity varices	-	Suprarenal and infrarenal IVC (18)	C4/10	C2/4	IVC stenting	
14	56/M	81/176/26.1	Bilateral lower extremity swelling and ulcerations	-	Infrarenal IVC and Left CIV (9)	C6/14	C5/7	Balloon angioplasty	IVC stenting and CDT
15	49/M	78/180/24.1	Bilateral lower extremity swelling and pain	Smoke and alcohol	Suprarenal and infrarenal IVC (15)	C4/8	C3/3	IVC stenting	-
16	71/F	62/157/25.2	Abdominal pain and bilateral lower extremity varices	-	Suprarenal IVC (7)	C4/9	C4/5	IVC stenting	-

Note: CIV, Common iliac vein.

All patients presented with symptoms associated with IVC occlusion. Specifically, 9 patients had abdominal wall varices, and 4 reported abdominal distension or pain. The severity of lower limb venous disease was assessed using the CEAP classification system (Eklof et al., 2004), with the following distribution: C1 in 2 patients, C2 in 2, C3 in 2, C4 in 4, and C6 in 6 patients.

All patients underwent endovascular treatment with a technical success rate of 100%. A total of 12 patients were treated via femoral vein access alone, while 4 underwent combined femoral and jugular vein access. Three patients received balloon angioplasty alone, of whom 2 required reintervention. The remaining 13 patients received stent placement during the initial procedure. In total, 27 stents were deployed in the IVC (26 self-expanding and 1 covered stent), and 10 stents were placed in the iliac veins. All patients experienced significant symptom relief following recanalization, resulting in a clinical improvement rate of 100%. The average hospital stay was 8.4 ± 1.7 days (range: 6–12 days).

### Endovascular treatment and complications in patients with IVC occlusion

**FIGURE 2:**
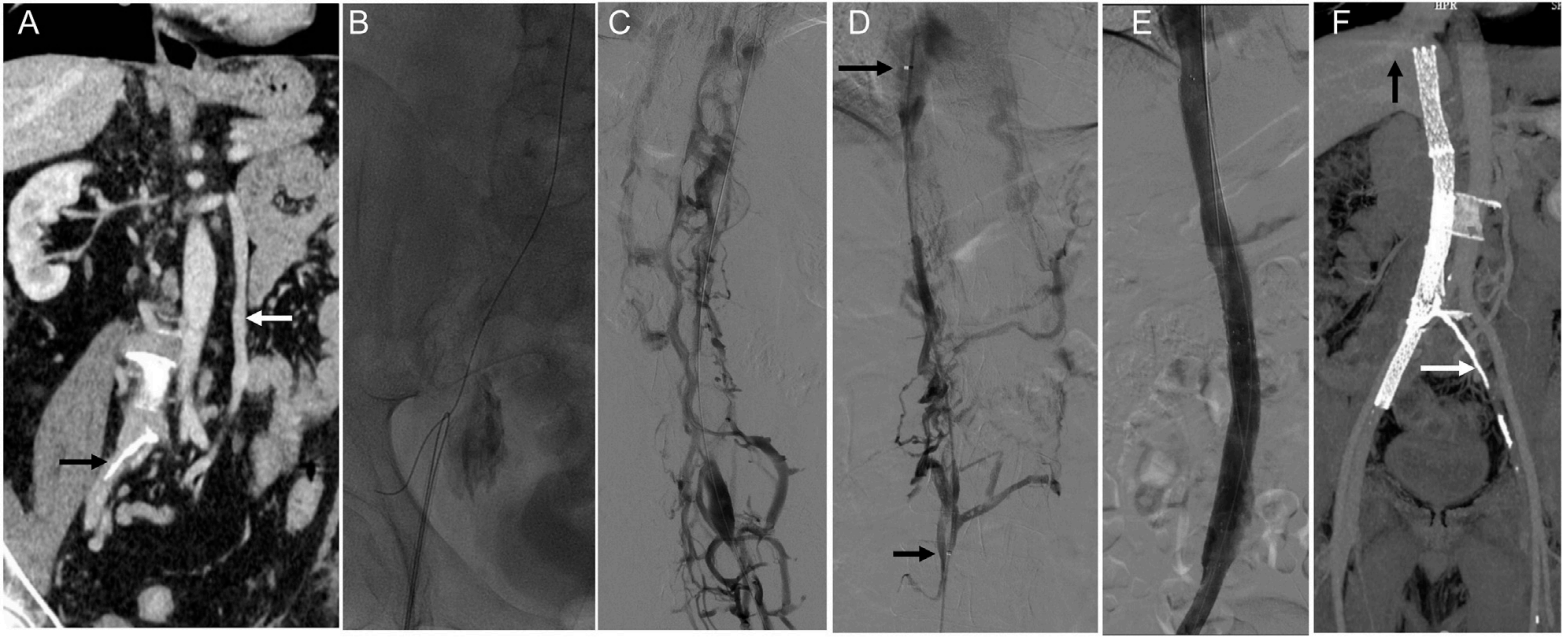
Innovative treatment and 1-year follow-up outcomes of IVC stenosis. Note: **(A)** A CT scan revealed a complete occlusion of the IVC, extending from the bilateral iliac veins to the entrance of the right atrium. The white arrow indicates the compensatory dilatation of the left lumbar vein, while the black arrow points out the calcified region of the iliac vein. **(B)** After unsuccessful attempts to access the occlusion via the femoral vein, a stiff wire and a guiding catheter were successfully inserted into the obstructed IVC through the right internal jugular vein. Subsequently, the wire was removed from the groin. **(C)** Femoral venography revealed the presence of abundant collateral circulation, which raised doubts about the correct placement of the wire in the IVC. **(D)** A thrombolysis catheter was successfully placed in the IVC, and the position of the catheter in the true lumen was confirmed through venography. The distance between the two arrows indicates the effective length of the thrombolysis catheter. **(E)** Gradual dilation of the IVC was performed using a balloon. Despite the dilation, the imaging results showed that the stenosis of the IVC still exceeded 50%. Therefore, four Wallstents and two Luminex stents were placed between the hepatic and right external iliac veins. After the stents were placed, subsequent imaging results demonstrated the restoration of patency in the IVC. **(F)** The one-year follow-up CT scan confirmed the IVC and right iliac vein patency with no residual stenosis. The black arrow in the image points to the hepatic vein, while the white arrow indicates the calcified region of the iliac vein.

Case 1: A patient with complete IVC occlusion continued to suffer from varicose veins and ulcers after 20 years of conservative treatment. Preoperative imaging revealed complete absence of the IVC and bilateral common iliac veins with calcification. Six stents were successfully deployed in the IVC and right iliac vein via combined femoral and jugular access. Although left iliac vein recanalization failed, right-sided reconstruction restored adequate hemodynamics. Postoperative follow-up showed complete ulcer healing and symptom resolution (Figure 2).

**FIGURE 3:**
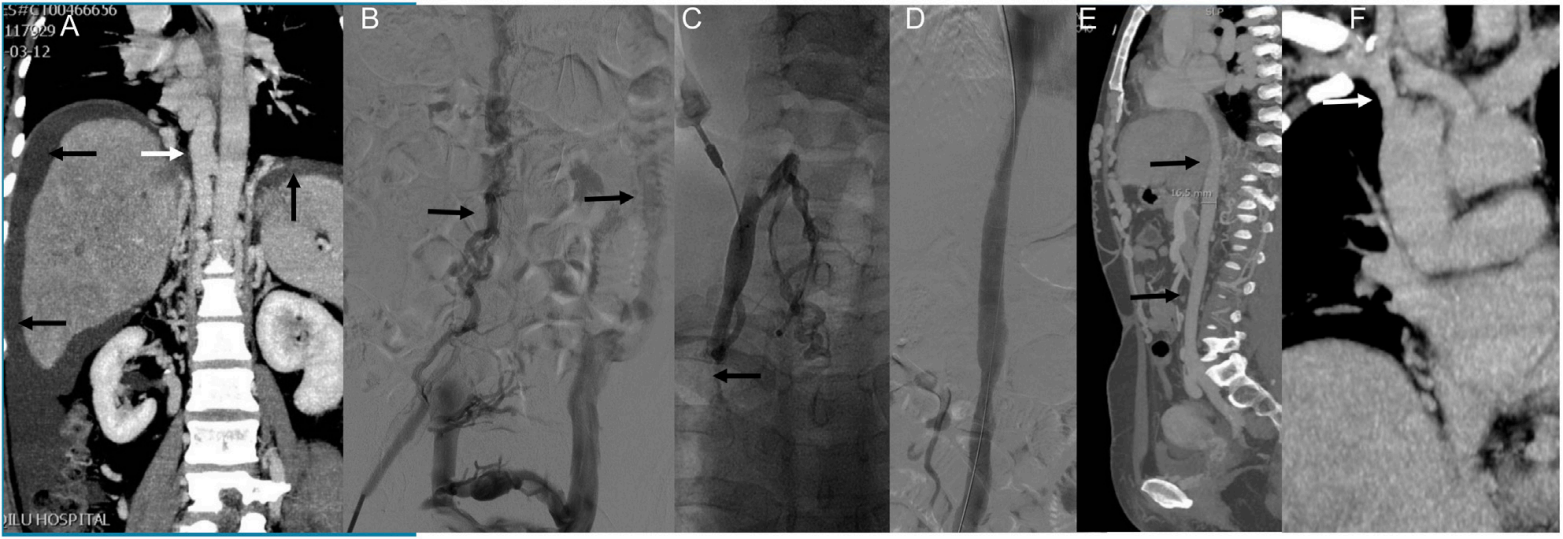
Innovative treatment and one-year follow-up of severe IVC occlusion. Note: Case 2 presented our study’s most extended medical history and severe clinical symptoms. The patient had been suffering from abdominal and bilateral lower limb varicose veins for over 20 years, with a right lower limb ulcer persisting for 10 years. The patient was admitted this time due to abdominal distension. **(A)** An enhanced CT scan revealed complete occlusion of the IVC, spanning from the atrial entrance to the bilateral common iliac veins and a significant amount of ascites. **(B)** Femoral venography showed complete occlusion of the IVC and bilateral common iliac veins. Compensatory lumbar veins were observed as the primary route for lower limb venous return (indicated by arrows). Attempts to pass a guidewire and supporting catheter through the IVC via the bilateral femoral veins were unsuccessful. **(C)** Subsequently, the right internal jugular vein was punctured, and contrast imaging revealed occlusion of the right brachiocephalic vein. Balloon angioplasty was performed to restore the patency of the right brachiocephalic vein, allowing the guidewire to pass through the IVC and the right common iliac vein via the right internal jugular vein channel. Gradual dilation of the IVC was then carried out using an 8–16 mm balloon. However, when a 16 mm balloon was used, the patient reported unbearable back pain, which prevented using an enormous balloon. **(D)** Following balloon dilation, the IVC became unobstructed and collateral circulation disappeared. **(E)** One year later, CT angiography revealed patency of the IVC (indicated by arrows) and the absence of ascites. **(F)** Additionally, the right brachiocephalic vein appeared unobstructed (indicated by arrows).

Case 2: This patient presented with long-standing abdominal and bilateral lower extremity varices and a 10-year history of right leg ulceration. CT imaging showed total IVC occlusion from the atrial inlet to both common iliac veins, with massive ascites (Figure 3A). Femoral venography confirmed complete IVC and iliac vein occlusion (Figure 3B). After failed attempts via femoral access, the right internal jugular vein was punctured. Balloon angioplasty reopened the occluded brachiocephalic vein, allowing guidewire passage to the IVC and iliac vein. The IVC was gradually dilated using 8–16 mm balloons, but further dilation was limited due to severe back pain (Figure 3C). The IVC achieved patency with disappearance of collateral flow (Figure 3D). At one-year follow-up, CT confirmed sustained IVC patency and complete resolution of ascites (Figures 3E,F).

**FIGURE 4:**
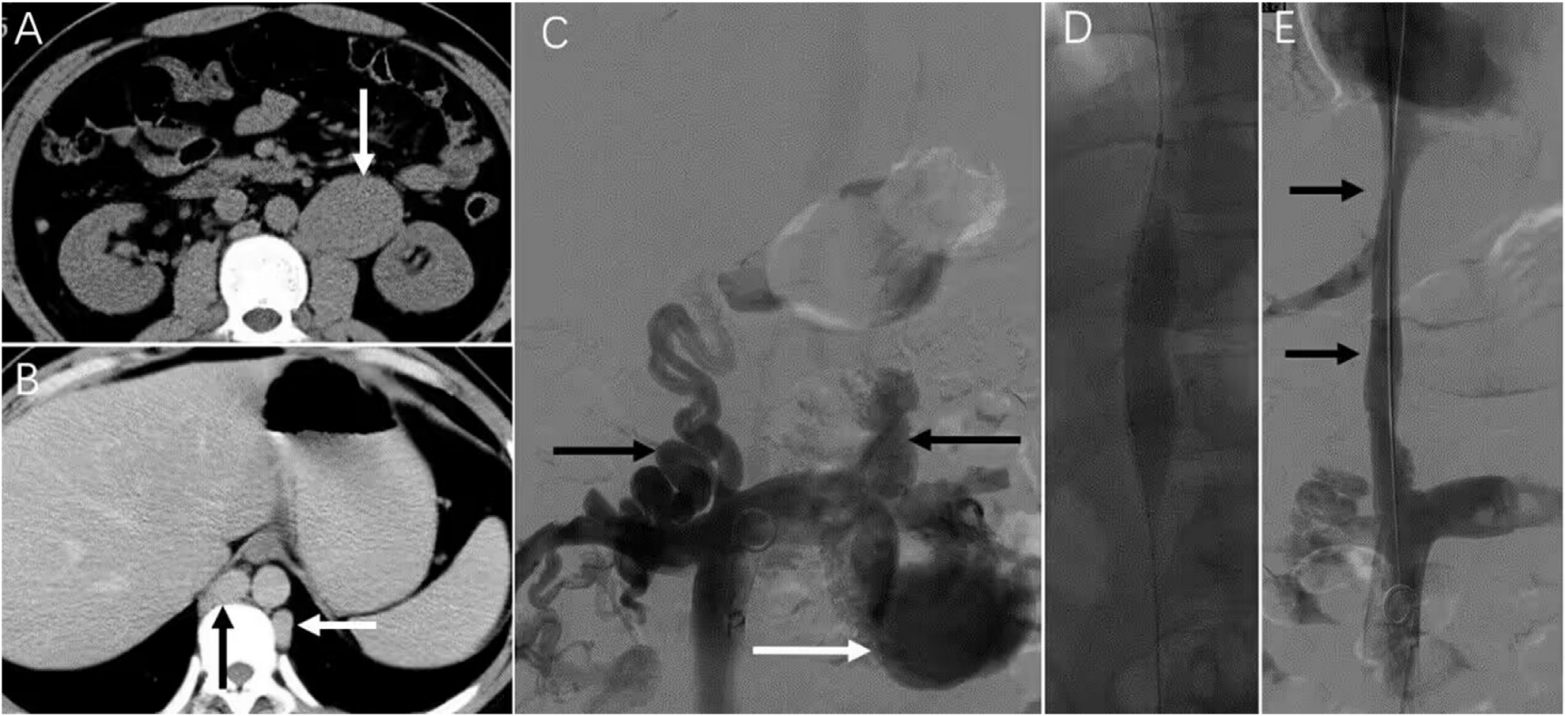
Serious complications that may be encountered in endovascular treatment. Note: The patient in case 3 presented with persistent abdominal distension for the past 6 months. **(A)** Upon a CT examination, a retroperitoneal tumor was suspected. **(B)** Further investigation through an enhanced CT scan revealed the presence of a left renal vein aneurysm and the absence of the IVC. The azygos and Hemiazygos vein displayed significant dilation (indicated by black and white arrowheads). **(C)** Vascular angiography was conducted, which confirmed the absence of the suprarenal IVC and the development of collateral circulation (indicated by a black arrowhead). A left renal vein aneurysm was also observed (white arrowhead). **(D)** Subsequently, dilation of the IVC was noted. **(E)** Following the vascular intervention, the IVC regained its normal patency (indicated by an arrow).

**FIGURE 5:**
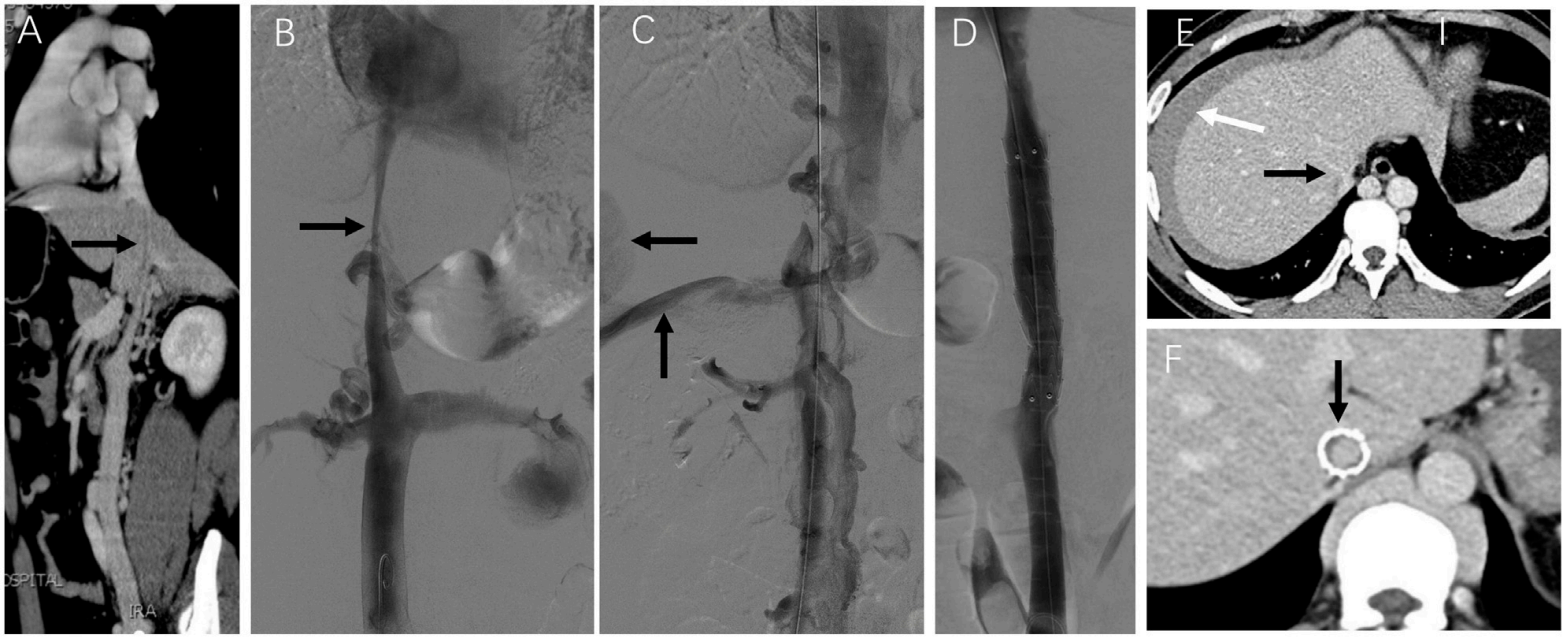
Emergency treatment and long-term observation of IVC stenosis. Note: After 20 months, the patient in case 3 experienced bloating once again. **(A)** Subsequent CT angiography performed 2 years later revealed the presence of severe stenosis in the IVC (indicated by arrows). **(B)** Despite dilatation using a 20 mm balloon, angiography still demonstrated considerable stenosis in the IVC (indicated by arrows). **(C)** During the dilation procedure using a more enormous 26 mm balloon, the patient reported experiencing intense abdominal and back pain. Following the angioplasty with the 26 mm balloon, there was a visible extravasation of contrast agent (indicated by arrows) and occlusion of the IVC due to abundant collateral circulation. The patient rapidly experienced a drop in blood pressure, which indicated a rupture in the IVC. **(D)** To address the rupture, a covered stent measuring 16*60 mm was successfully placed within the IVC between the hepatic and renal veins. This placement restored the patency of the IVC without any contrast agent leakage. The patient’s condition improved following transfusion and fluid replacement therapy, resulting in discharge from the hospital on the 7th day after the surgery. **(E)** A CT scan 7 days later showed unobstructed hepatic veins and IVC (indicated by black arrows), with an intra-abdominal hematoma (indicated by the white arrow). **(F)** In the first year following stent placement, a CT scan confirmed the patency of the stented area in the IVC with no signs of stenosis (indicated by arrows).

Case 3: A patient presented with 6-month abdominal distension and was initially suspected to have a retroperitoneal tumor based on CT imaging (Figure 4A). Enhanced CT revealed a left renal vein aneurysm and absence of the suprarenal IVC, with significant dilation of the azygos (black arrow) and hemiazygos veins (white arrow) (Figure 4B). Venography further confirmed suprarenal IVC absence and extensive collateral circulation (Figure 4C, black arrow); the renal vein aneurysm was also visualized (white arrow). Following guidewire passage, the IVC demonstrated segmental expansion under balloon dilation (Figure 4D). Final angiography showed successful recanalization and restoration of normal flow through the IVC (Figure 4E). However, 20 months later, CT showed severe restenosis of the IVC (Figure 5A). Despite dilation with a 20 mm balloon (Figure 5B), further angioplasty with a 26 mm balloon led to IVC rupture, indicated by contrast extravasation and hypotension (Figure 5C). Emergency placement of a covered stent between the hepatic and renal veins controlled the bleeding (Figure 5D). Follow-up imaging confirmed restored patency without leakage (Figures 5E,F).

Additionally, three patients developed access site hematomas, all resolved with compression therapy. These cases highlight the technical complexity, potential complications, and clinical value of timely endovascular intervention in IVC occlusion.

### Clinical outcomes and complications of endovascular treatment for IVC occlusion in long-term follow-up

**FIGURE 6:**
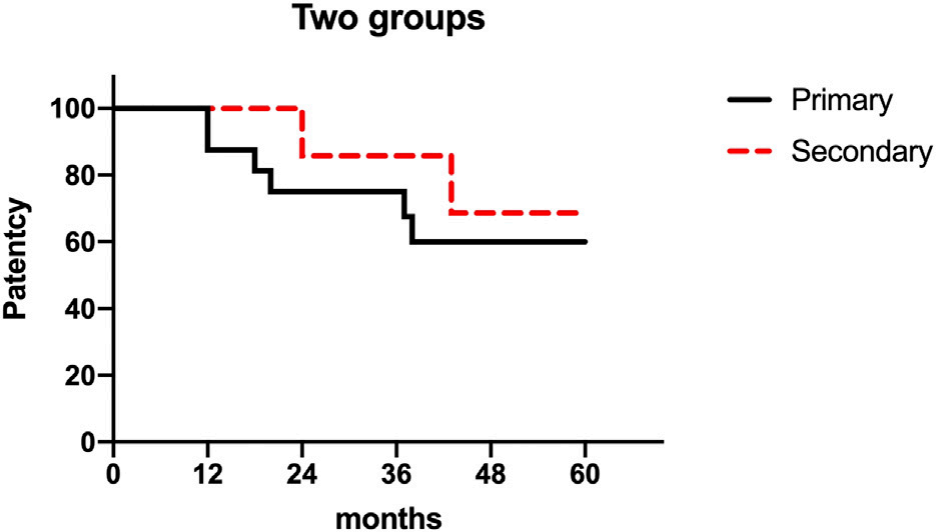
A study on cumulative first and second patency rates after 16 cases of venous reconstruction surgery. Note: The figure presented displays the cumulative rates of primary and secondary patency following 16 instances of venous reconstruction surgery. The graph’s measurements are 301 × 187 mm, and its resolution is 96 × 96 DPI.

The mean follow-up duration was 47.4 months (range: 18–81 months), with complete follow-up data available for all patients. The primary patency rate at 36 months was 75%, and the secondary patency rate reached 87.5% (Figure 6). No procedure-related deaths occurred.

Clinically, all patients experienced significant relief from abdominal distension and pain. Among nine patients with abdominal varices, six reported improvement. All patients received standardized anticoagulation and used compression stockings as instructed.

The median VCSS score improved significantly from 9.3 ± 4.6 to 4.4 ± 2.4 after treatment (P < 0.01). All six patients with active venous ulcers achieved complete healing. One patient (Case 12) experienced ulcer recurrence at 18 months, which resolved after reintervention.

Complications included acute venous thrombosis in Case 14 at 38 months, managed with catheter-directed thrombolysis and stenting. Stent occlusion was observed in Case 6 and Case 15 at 12 and 24 months, respectively, but no reintervention was required due to mild symptoms. Two other patients had mild restenosis (<30%) without symptoms and remained under observation.

No severe complications such as renal/hepatic dysfunction, pulmonary embolism, or heart failure were observed during follow-up. These findings support the long-term safety and efficacy of endovascular treatment for IVC occlusion.

### Quantitative analysis results of literature review

To investigate the progress of endovascular treatment for primary long-segment IVC occlusion, relevant literature was reviewed. The screening process is summarized in Supplementary Figure S1. After initial filtering, 78 studies were included; approximately 80% focused on IVC occlusion caused by malignancy or thrombosis, while only 20% addressed primary IVC occlusion, with long-segment cases being particularly rare. Eight retrospective studies or case reports related to endovascular treatment were selected for further analysis.

All included articles were published in peer-reviewed journals with full-text availability. Quality assessment results are shown in Supplementary Figure S2, indicating a low risk of bias in indicator selection.

Table 2 presents the baseline characteristics of 8 included studies involving 210 diagnosed patients with primary IVC occlusion. The main treatment methods employed in these 8 studies were vascular angioplasty and stent placement, which demonstrated favorable short-term or long-term treatment outcomes. Only 2 studies included patients with long-segment IVC occlusion. Due to the limited number of cases in the study by Bi et al. (2022), accurate information regarding the prevalence of primary long-segment occlusion of the IVC could not be obtained. However, the study collected literature from 1992 to 2022, indicating the rarity of primary long-segment occlusion of the IVC. Notably, advancements in endovascular intervention have improved the treatment effectiveness of primary IVC occlusion over time. Endovascular stent placement has emerged as a safe treatment option, particularly for patients who do not exhibit sustained clinical improvement.

**TABLE 2:** Baseline characteristics of patients included in the study (n = 8).

Study	Year	Age	Research type	Patients (n)	Lesion type	Treatment	Short-term or long-term improvements
Bi et al. (2022)	2022	50.4 (23–72)	retrospective study (2012–2019)	34	IVC and hepatic vein involvement; Isolated IVC involvement; Segmental obstruction (including **long segmental obstruction**); Membranous obstruction; IVC thrombosis involvement	A jugular-femoral venous route for recanalization	Technical success of IVC recanalization was achieved in remaining 33 (97.1%) patients. The 1-year, 3-year, 5-year primary patency rates were 85.9%, 76.4% and 70.0%, respectively. The 5-year secondary patency rate was 96.8%
Klepitsch et al. (2020)	2020	47 (21–88)	retrospective study (2010–2019)	13	-	Gianturco tracheobronchial Z-stents	Technical success was achieved in all patients. Longer-term follow-up was available in 5 patients with non-malignant occlusions and all (5/5) demonstrated maintained patency of IVC stents
Erben et al. (2018)	2018	43 (17–83)	retrospective study (2001–2014)	66	long-standing chronic venous obstruction	Sequential angioplasty and stent placement	Estimated patency rates at 36 months were >85%, and clinical outcomes were excellent
Grøtta et al. (2017)	2017	39.9 (15–61)	retrospective study (2010–2015)	20	chronic obstruction of the IVC	Stent placement and catheter directed thrombolysis	Primary patency after 24 months was 67% and secondary patency 83%. There were no peri-procedural or long-term complications
Ceretti et al. (1997)	1997	23/38	case report	2	-	Self-expandable metallic stents followed by portosystemic shunt	At 14 months of follow-up the patient enjoys good health
Yang et al. (1996)	1996	35.6 (16–56)	retrospective study (1988–1996)	42	membranous obstruction	Percutaneous transluminal balloon angioplasty (PTBA)	All 38 patients who successfully underwent PTBA showed marked symptomatic improvement
Xu et al. (1996)	1996	36.3 (20–56)	retrospective study (1988–1992)	32	membranous obstruction; segmental stenosis or occlusion	Percutaneous transluminal angioplasty (PTA) and expandable metallic stent (EMS) placement	PTA alone produces excellent short-term results and about 50% sustained patency after 2 years in patients with BCS; therefore it should remain the procedure of first choice. Stents should be reserved for primary or secondary PTA failures
Ishiguchi et al. (1992)	1992	42	case report	1	**long segmental thrombotic obstruction of the IVC**	Combination of local thrombolytic therapy, balloon angioplasty, and placement of Gianturco expandable metallic stents	The patient was successfully treated

Note: Bold text indicates long segmental thrombotic obstruction of the IVC, where IVC stands for inferior vena cava.

## Discussion

Long-segment occlusion of the IVC is a rare condition that can be caused by various factors, including hypercoagulable disorders, tumors, IVC injury, inflammation, congenital disabilities, or Budd-Chiari syndrome (Harris, et al., 1976; Razavi et al., 2000). However, the cause of IVC occlusion in some patients remains unclear in this study. Thrombosis and tumors have been ruled out as the main contributors in all cases. Some research suggests that congenital IVC atresia may play a role (Gil et al., 2006). It is important to note that congenital IVC atresia is different from IVC occlusion, and it is a scarce condition with an incidence rate of 0.2%–0.6%, often associated with congenital heart disease (Yun et al., 2004; Gil et al., 2006). Although DVT is a common clinical manifestation in this series, none of the patients in this study had a history or evidence of DVT. About 50% of patients with congenital IVC atresia are asymptomatic due to alternative circulatory pathways such as retroperitoneal, paravertebral, intraspinal, and left gonadal veins (Okuda et al., 1998). Despite collateral circulation in all cases, patients display varying symptoms due to inadequate compensatory capacity. De Maeseneer et al. reported an average age of diagnosis of 29 years among 35 patients with congenital IVC agenesis (De Maeseneer et al., 2013), which differs from the cases in our study. Compared to our sample, patients with congenital IVC atresia are generally younger and face more difficulty in reopening the IVC.

Previous research has revealed that the etiology of IVC occlusion may vary depending on the specific segment affected. In the case of suprarenal IVC anomalies, Shah NL et al. (Shah et al., 1996) reported that approximately 90% of these abnormalities are congenital, with only 6% of patients exhibiting congenital abnormalities involved in the renal or infrarenal IVC. On the other hand, some researchers suggest that the absence of infrarenal IVC is not a result of embryogenesis but rather perinatal thrombosis (d'Archambeau et al., 1990; Salgado Ordonez et al., 1998; Alicioglu et al., 2009). However, it is currently unclear whether perinatal thrombosis occurred in our four patients with infrarenal IVC occlusion.

Individuals with long-segment IVC occlusion commonly experience various symptoms, including leg swelling, pain, varicose veins, abdominal distention, and abdominal venous dilatation (Koc and Oguzkurt, 2007). Prolonged occlusion of venous outflow can lead to venous hypertension and valve dysfunction. In our study, all patients exhibited varying degrees of CVI, with 12 cases having a clinical CEAP score of ≥3. Following treatment, 68.6% (11/16) of patients achieved significant relief from their symptoms, while 83.3% (10/12) experienced noticeable improvement. Following IVC recanalization, all patients were prescribed compression therapy with elastic stockings due to persistent venous valve dysfunction in the lower extremities caused by chronic occlusion. This approach aims to improve lower limb venous return and prevent chronic venous insufficiency.

In this case series, all patients with lower limb ulcers had concurrent iliac vein occlusion (Cases 1, 2, 4, 7, 12, 14), whereas those with patent iliac veins exhibited relatively mild lower limb symptoms. These findings highlight the importance of iliac vein patency in maintaining effective venous return in the setting of IVC occlusion. In such cases, collateral pathways may develop to redirect lower limb venous flow to the superior vena cava. These alternate routes include the lumbar ascending and azygos veins (Raju et al., 2006). However, when both the iliac vein and IVC are obstructed concurrently, compensatory mechanisms may prove insufficient, leading to the development of severe lower limb symptoms.

Some patients with long-segment IVC occlusion, especially those with congenital anomalies, may remain asymptomatic. In such cases, surgical intervention is generally not indicated. However, previous studies have reported that asymptomatic individuals with IVC occlusion may later develop acute deep vein thrombosis (Yun et al., 2004; Raju et al., 2006). Consequently, long-term anticoagulation is recommended for asymptomatic patients with IVC occlusion. However, conservative management alone may be insufficient, as demonstrated by the persistent ulcers observed in several cases. For symptomatic patients, surgical intervention should be considered. In recent years, endovascular treatment has become the preferred approach due to its minimally invasive nature and favorable outcomes, whereas open surgery is limited by higher early complication rates and lower patency (Jost et al., 2001; Hartung et al., 2005; Garg et al., 2011; DeRubertis et al., 2013). Although percutaneous recanalization of IVC occlusion is challenging, we achieved successful outcomes in all 16 patients.

Nonetheless, there are limited reports on the endovascular treatment of complete IVC occlusion (Adam et al., 2015; Zhang et al., 2015). In a study by Zhang et al. (2015), they successfully treated five patients with complete IVC occlusion using endovascular stents. Our study encountered two patients with complete IVC occlusion, one with simultaneous occlusion of the bilateral iliac veins and the right brachiocephalic vein. However, we managed to recanalize the IVC, iliac vein and right brachiocephalic vein through balloon dilation (Figure 3). Based on our experience, the treatment effect of IVC long-segment occlusion with balloon angioplasty alone is not optimal due to extensive recoil. Nevertheless, the follow-up results at 3 years exceeded our expectations. This is the first report describing the successful recanalization of complete IVC occlusion using balloon angioplasty alone.

Anticoagulation therapy is a crucial treatment approach for patients following IVC recanalization. However, there is a need for clear guidelines concerning the appropriate duration of treatment. Existing literature suggests that individuals who undergo IVC stenting should receive oral anticoagulation therapy for 6–12 months (de Graaf et al., 2015). Some experts even advocate for lifelong antiplatelet therapy for all patients (Chick et al., 2017; Murphy et al., 2017). Furthermore, studies have indicated that individuals with congenital IVC occlusion should undergo lifelong anticoagulation therapy (Kearon et al., 2012; De Maeseneer et al., 2013). Given the ambiguous cause of IVC occlusion in this study, it is recommended that all patients receive lifelong anticoagulation therapy to minimize the risk of IVC and stent thrombosis.

Among the cases examined in this study, 5 instances of recurrent IVC occlusion or stenosis were identified, with 2 cases attributed to venous recoil and 3 cases caused by thrombosis. These 3 patients underwent a total of 4 repeat interventions. Nevertheless, the study reported a relatively satisfactory midterm patency rate, with 87.5% (14/16) of patients maintaining IVC patency during the final follow-up. Notably, long-segment IVC occlusion was found to be closely associated with involvement of the hepatic and renal veins. Specifically, 12 patients had lesions above the renal veins, and 4 had lesions above the hepatic veins. Despite this, all patients exhibited normal renal function, potentially due to the development of abundant collateral circulation during chronic renal vein occlusion, such as the left-sided and accessory hemiazygos veins and lumbar veins, thereby sustaining renal function. Consequently, none of the patients required renal vein reconstruction.

The surgical risk is low for patients undergoing endovascular intervention for long-segment occlusion of the IVC, and the success rate is high, as demonstrated in this study. The postoperative complications were minimal, with no reported deaths. However, one severe complication was observed, namely, IVC rupture caused by balloon dilation. Thankfully, this was promptly managed using a covered stent, resulting in no long-term consequences for the patient.

To appropriately diagnose IVC occlusion, it is crucial to utilize enhanced CT and color Doppler ultrasound examinations. Enhanced CT provides a clear visualization of the IVC and its branches, while color Doppler ultrasound aids in detecting lower limb thrombosis. However, it is essential to note that ultrasound findings for patients with IVC occlusion can be inconsistent, requiring an experienced operator for accurate interpretation. Consequently, enhanced CT examinations were conducted before and after the surgical procedure for all cases in this study.

Current literature indicates that endovascular reconstruction of the IVC is typically performed under general anesthesia or local anesthesia combined with sedation (Murphy et al., 2017; Erben et al., 2018; McDevitt et al., 2019). However, in this study, all procedures were successfully completed under local anesthesia alone, without the use of sedation. This approach offers several advantages: it significantly reduces the risk of anesthesia-related complications, particularly in patients with multiple comorbidities; it allows patients to remain conscious during the procedure, enabling real-time feedback and immediate adjustment by the operator; and it avoids potential respiratory depression associated with sedative agents, thereby facilitating faster postoperative recovery without the need for anesthetic monitoring. These findings suggest that, in the hands of an experienced interventional team, local anesthesia can serve as a safe and effective option for IVC endovascular reconstruction and may offer a new evidence-based alternative for optimizing anesthesia protocols in such procedures.

This study has certain limitations. The relatively small sample size may affect the generalizability of the results. Additionally, the retrospective single-center design may limit data completeness due to variability in clinical records and follow-up availability. Furthermore, as primary long-segment IVC occlusion is a rare condition, conducting large-scale prospective randomized controlled trials remains a substantial challenge. Therefore, further multicenter, prospective studies are warranted to validate these findings.

Despite these limitations, this study provides valuable clinical insights. Through systematic review and case analysis, it presents one of the largest case series to date on endovascular treatment for primary long-segment IVC occlusion. The findings demonstrate favorable safety and feasibility outcomes of IVC reconstruction under local anesthesia and offer practical evidence to support optimization of surgical and anesthetic strategies. In addition, the results further underscore the importance of standardized compression therapy and timely intervention in the management of IVC occlusion.

## Data Availability

The raw data supporting the conclusions of this article will be made available by the authors, without undue reservation.

## Supplementary material

SUPPLEMENTARY FIGURE S1 PRISMA flow-chart.

SUPPLEMENTARY FIGURE S2 Quality assessment of included literature. Note: In risk categorization, the color red is indicative of a high level of risk, whereas green denotes a low level of risk. On the other hand, yellow is assigned to circumstances where the level of risk remains uncertain or cannot be determined.
