# Supplementary figures and images for: Endovascular management of primary long-segment inferior vena cava occlusion: treatment strategies and clinical outcomes
Source: Front Pharmacol. 2025 Jun 6;16:1512157. doi: 10.3389/fphar.2025.1512157 (PMC12179148; doi:10.3389/fphar.2025.1512157)

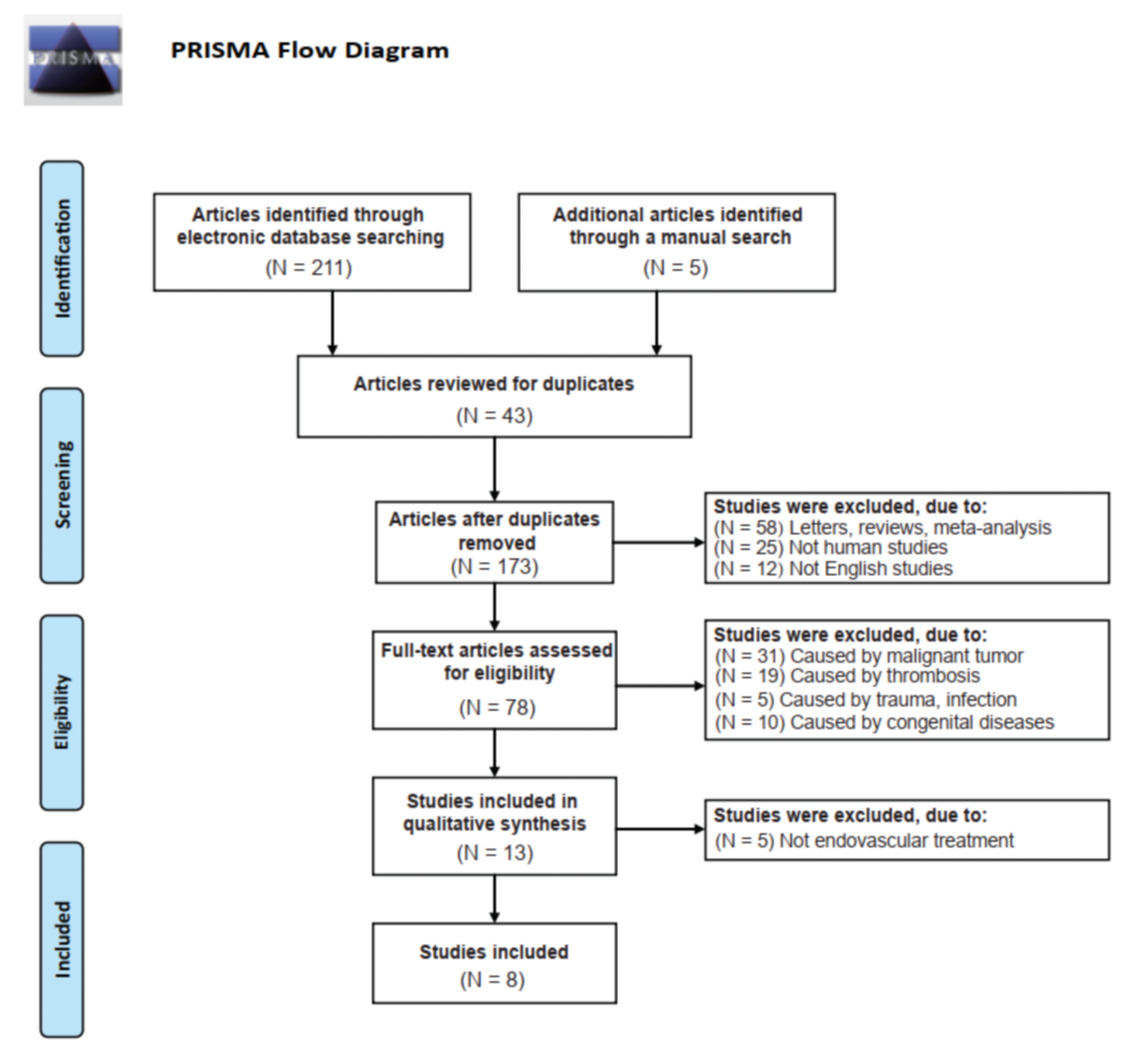

Supplement: Supplementary file 1 [file Image1.jpeg]

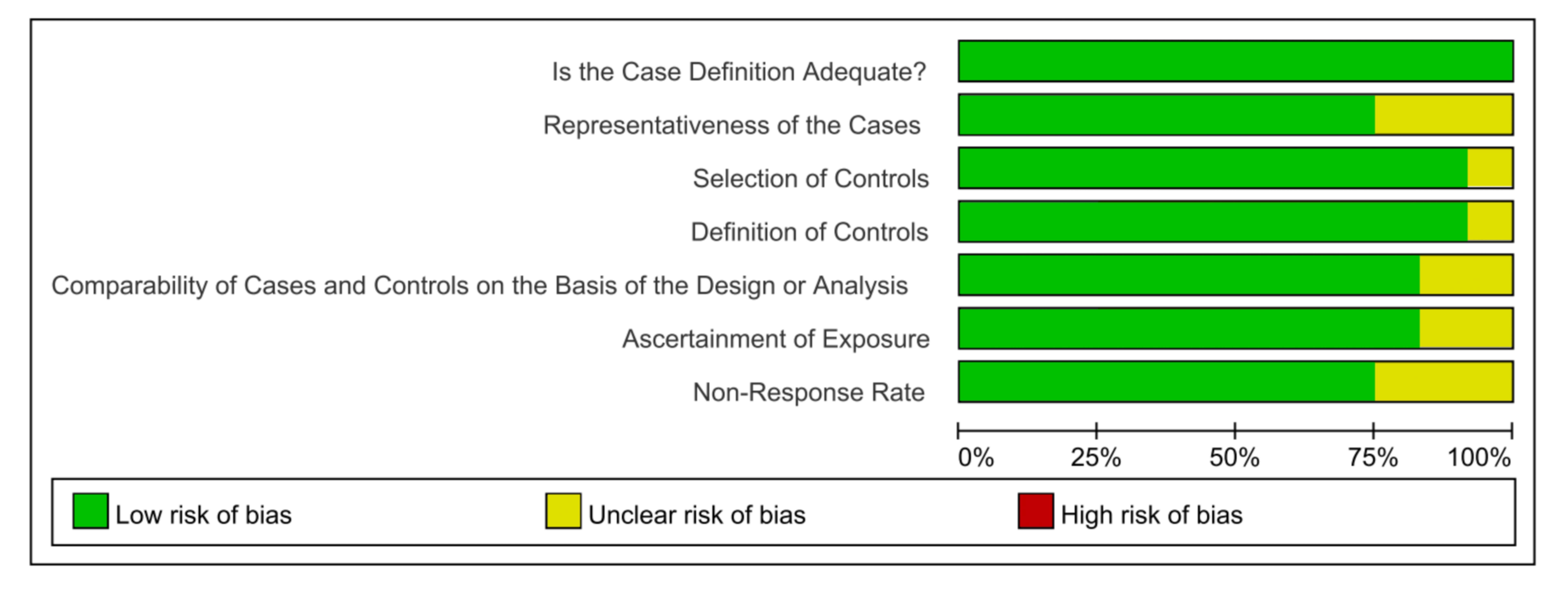

Supplement: Supplementary file 2 [file Image2.jpeg]
